# A qualitative study of the development and utilization of health facility-based immunization microplans in Uganda

**DOI:** 10.1186/s12961-021-00708-y

**Published:** 2021-08-11

**Authors:** David Kaawa Mafigiri, Constance Iradukunda, Catherine Atumanya, Michael Odie, Arielle Mancuso, Nhan Tran, Janet McGrath, Henry Luzze

**Affiliations:** 1 Center for Social Sciences Research on AIDS, School of Social Sciences, Makerere University, P.O. Box 7062, Kampala, Uganda; 2 Alliance for Health Policy and Systems Research, WHO Headquarters, Geneva, Switzerland; 3 Department of Anthropology, Center for Social Sciences Research on AIDS, Case Western Reserve University, Cleveland, OH USA; 4 Uganda Ministry of Health/UNEPI, P.O. Box 7272, Kampala, Uganda

**Keywords:** Microplan, Utilization, Routine immunization, Qualitative study, Uganda

## Abstract

**Background:**

In 2006, Uganda adopted the Reaching Every District strategy with the goal of attaining at least 80% coverage for routine immunizations in every district. The development and utilization of health facility/district immunization microplans is the key to the strategy. A number of reports have shown suboptimal development and use of microplans in Uganda. This study explores factors associated with suboptimal development and use of microplans in two districts in Uganda to pinpoint challenges encountered during the microplanning process.

**Methods:**

A qualitative study was conducted comparing two districts: Kapchorwa, with low immunization coverage, and Luwero with high immunization coverage. Data were collected through multilevel observation of health facilities, planning sessions and planning meetings; records review of microplans, micromaps and meeting minutes; 57 interviews with health workers at the ministry level and lower-level health facility workers. Data were analysed using NVivo 8 qualitative text analysis software. Transcripts were coded, and memos and display matrices were developed to examine the process of developing and utilizing microplans, including experiences of health workers (implementers).

**Results:**

Three key findings emerged from this study. First, there are significant knowledge gaps with regard to the microplanning process among health workers at all levels (community and district health facility and nationally). Limited knowledge about communities and programme catchment areas greatly hinders the planning process by limiting the ability to identify hard-to-reach areas and to prioritize areas according to need. Secondly, the microplanning tool is bulky and complex. Finally, microplanning is being implemented in the context of already overtasked health personnel who have to conduct several other activities as part of their daily routines.

**Conclusions:**

In order to achieve quality improvement as outlined in the Reaching Every District campaign, the microplanning process should be revised. Health workers’ misunderstanding and limited knowledge about the microplanning process, especially at peripheral health facilities, coupled with the complex, bulky nature of the microplanning tool, reduces the effectiveness of microplanning in improving routine immunization in Uganda. This study reveals the need to reduce the complexity of the tool and to identify ways to train and support workers in the use of the revised tool, including support in incorporating the microplanning process into their busy schedules.

## Background

Routine immunization is the backbone of immunization as a public health tool [1]—a fundamental service intended to reach all children with multiple doses of vaccines. In 2002, WHO developed the Reaching Every District (RED) strategy which aimed to improve vaccination coverage globally. Following the introduction of the RED approach, routine immunization coverage improved in many low-income countries. However, coverage has stalled since 2008–2009, and globally as of 2017 an estimated 19.9 million infants had not been reached by routine immunization services [2].

In 2006, Uganda adopted the RED strategy with the goal of attaining at least 80% coverage for routine immunizations in every district across the country. In Uganda, the RED strategy evolved, as it has in many countries, to become Reaching Every Community/Child (REC) [1, 3]. The approach proposes five operational strategies: (1) Re-establishing health outreach where it has been dismantled. Outreach is any strategy that requires health facility staff to leave their facility to deliver immunization. Ideally, a minimum of five contacts per year is required to fully immunize an infant in Uganda. (2) Supportive supervision, defined as provision of regular onsite training to health workers. The supervision helps to solve problems locally and follow up supply and resource issues. (3) Linking services with communities so as to meet specific local needs. The community should be encouraged to take ownership of planning and service delivery. (4) Monitoring and use of data for action. This involves timely collection of data at the facility and district levels and use of the data to solve problems. (5) Planning and management of resources.

To operationalize the REC strategy, a health facility/district microplanning tool was developed to guide health workers in planning and delivering immunization services [1]. An effective microplan aims to reach all target populations with immunization services as recommended in the vaccination schedule. The microplan should be based upon a local situation analysis which involves every health facility, and through them the community that they serve. It includes actions for improving coverage and quality of services. REC microplans have well-defined catchment areas and realistic local solutions for operational, economic, political and social barriers to immunization [1]. The microplan describes the demographics of the health facility catchment area by listing villages and parishes (a parish is an organizing administrative structure comprising a number of villages along which service delivery is planned; several villages make up a parish, and several parishes make up a county and district) in the catchment area, total population per village and the target populations (children under 1 year, children under 5 years, pregnant women) [1]. It includes a sketch of the health facility catchment area with all routine immunization points (both static and outreaches) and geographical features such as rivers, swamps and mountains. Other topical areas the microplan captures are a mapping of immunization service points, both static and outreaches, showing how villages in the catchment area are served. Additionally, the microplan includes documentation on performance monitoring and dropout rate monitoring charts that are updated monthly. The microplan also has a section on social mapping indicating stakeholders and partners in villages and parishes. Finally, there is a section showing the plans and budget needed for implementation of the microplan.

Despite having adopted the RED strategy in 2006, by 2015, only 40 of the 112 districts had updated microplans. No systematic assessment had been conducted to assess underlying factors contributing to suboptimal development and use of microplans in Uganda. This study examined the process and challenges of developing and utilizing microplans for improving immunization outcomes in Uganda. In addition, the study identified gaps and explored strategies to improve the microplanning process in two districts in Uganda. By comparing a “high-performing” and “low-performing” district, the study was positioned to better understand how the development and implementation of the microplan process unfolded and the associated barriers to its adoption. The knowledge obtained from this work suggests strategies to address challenges confronting the microplanning process, with the overall aim of improving immunization rates at health facility and district levels.

## Methods

### Design and setting

This qualitative study used multiple methods to examine the process of developing and employing microplans in the districts of Luwero (with high immunization coverage) and Kapchorwa (low coverage) in Uganda [4]. Luwero district is located in Central Uganda, approximately 75 km (47 miles) by road north of Kampala, the capital city. Luwero is covered with savannah vegetation and characterized by a tropical climate. Agriculture is the main economic activity, with coffee and bananas constituting the major cash crops. Kapchorwa district is located in Eastern Uganda 287 km east of Kampala. Kapchorwa is generally hilly, with steep slopes and valleys and several rivers flowing from Mount Elgon. As such, Kapchorwa is covered with mixed mountainous forests and less open savannah compared to Luwero. Kapchorwa is sparsely populated due to both the terrain, which makes it more prone to rock- and landslides, and a history of insecurity arising from cattle rustling practices. The main economic activity is agriculture, with coffee, millet and potatoes constituting the majority of crops farmed and traded. A majority of the population in both districts is made up of children. Although several districts at the time of the study had rolled out the RED programme, both Luwero and Kapchorwa were among the first districts to participate in the pilot programme when the intervention was tested. As such, they had both undergone much more exposure to the use of microplans. Both districts were purposively selected given their unique setting and characteristics to represent the variability of population characteristics in Uganda. Methods included multilevel observation of health facilities, planning sessions and planning meetings; records reviews of microplans, micromaps and meeting minutes; and interviews. The 2014 WHO/United Nations Children’s Fund (UNICEF) joint report states that the national immunization coverage in Uganda, as measured by the third dose of diphtheria, pertussis, tetanus, hepatitis B and *Haemophilus* influenza vaccine (DPT3), was 100% [5]. Luwero district, with a total population of 467,466, 20,101 children under 1 year, and 65 health facilities, reported DPT3 coverage of 115%. Kapchorwa district, with a population of 121,163, 5210 children under 1 year, and 20 health facilities, had DPT3 coverage of only 66% [5]. Luwero is more densely populated and has many more health facilities than Kapchorwa; the study purposively selected nearly all facilities in Kapchorwa to increase the representation of experiences given the vast expanse and variability within Kapchorwa district.

Health facilities in Uganda are classified into seven levels based on the services they provide and the catchment area they serve. The health facilities are designated as health centre levels I to IV (HC I–IV), general hospital, and regional and national referral hospitals [6]. Immunization in Uganda is part of the integrated minimum health care package delivered by local governments at the district level. Therefore, responsibility for the delivery of health services, including immunization, rests with local district governments, each operating under the mandates of the national health system. At the district level, the health system is composed of different structures, where the lowest level within the community is HC I (composed of village health team members), followed by HC II, HC III, HC IV and general hospital, respectively. This ranking is based on population size in the catchment area, beginning with HC I facilities, where each village health team serves approximately 1000 persons, or 25 households in less densely populated areas. This ranking corresponds to the population size reflected by the local administrative structures and covers similarly larger populations as one moves along the different levels of hospitals and administrative structures. At present, under the RED strategy, all health facilities from HC II and above in the district are expected to have community-based microplans for immunization. Study participants were selected to represent the four levels constituting a district catchment area. Notably, some participants at the district level also represented the national level, as they serve in policy and planning at the national ministry level by virtue of their offices held at the district level. Fifty-two public health facilities in the two districts (*n* = 17 in Kapchorwa) were examined in this study for microplan utilization patterns using both in-depth interviews and review of documents (facility records such as maps, meeting minutes and immunization record books). Notably, nine of the health facilities examined (*n* = 4 in Kapchorwa) were owned by private nongovernmental organizations (NGOs) that engage in immunization activities implemented by the Ministry of Health. Health facilities were also purposively selected for the study, aiming to cover both rural and urban settings in both districts. In both districts, the study focused on different stakeholders at the selected sites, namely (1) health workers at both public and private health facilities involved in immunization activities, including managers of the Expanded Programme on Immunization (EPI) at the national and district levels, health facility managers and health workers at the facility level; (2) community members, particularly those involved in child care, who may be responsible for seeking immunization services in the two regions including district-level administrators, chief administrative officers and district local council chairpersons, and area members of Parliament who influence immunization programmes.

### Data collection

The study relied on purposive sampling to determine the participants to interview. Social science research assistants (RAs) with prior experience in qualitative data collection techniques conducted 57 key informant interviews (*n* = 17 in Kapchorwa and *n* = 40 in Luwero) with health workers responsible for immunization services. Official letters were written by the principal investigators (PI) informing the districts of the project and introducing the study team. The district health personnel were also provided with copies of Makerere University’s ethical clearance letters that were attached to the PI’s introductory letter. A designated member of the district health team (DHT) in each district worked with the project team to identify participants from the health facilities and the community within the catchment area of a particular health facility. Key informants were approached by an RA and invited to participate. Enrolment of participants and data collection continued until saturation was reached. Interviews focused on several domains: experiences, knowledge of and practices during the process of developing the microplans, and successes and challenges encountered during the microplanning process. Two study investigators (DKM and HL) reviewed health facility records including immunization monitoring charts, catchment area maps, and outreach and district health office support supervision notes. Review of this data generated comparative data on level of knowledge and key landmarks such as critical events (social mobilization activities), individuals or documents utilized during implementation of microplans. During data collection, all study investigators (DKM, HL and JWM) monitored all data collection processes to ensure that the process complied with the study’s ethical and quality requirements.

### Data management and analysis

All interviews and field notes were transcribed verbatim and translated to English by the RAs. The data were then analysed using NVivo 8 qualitative text analysis software [7]. Transcripts were read through so that the researchers were familiar with the data, and then related categories were sourced out. For each category, we identified a number of codes, where each served as an indexing or measurement device to assign values to the text and to help organize the data. These data were first coded by two RAs and reviewed by one study investigator (DKM) for uniformity before all coding was complete. A codebook was developed and pilot-tested on five transcripts from key informant interviews. Topical and thematic codes were then applied to all transcripts and field notes following a content-driven theme approach [8–10]. Codes were synthesized into categories and themes, while variables were compared across levels of analysis. Findings from interview data were corroborated with summaries generated from review of various relevant documents and notes from observations made at health facilities. This process was carried out to ensure accuracy and to increase the validity of emerging themes.

## Results

Fifty-seven participants were drawn from different health facilities across the two districts, including HC I/II, HC III and HC IV facilities and hospitals. Table 1 illustrates the type of respondents by their job title, facility level and district.

**Table 1:** Type of health workers interviewed, by facility and district (*N* = 57)

	Luwero (*N* = 40)	Kapchorwa (*N* = 17)
*Job title*		
Administrative staff	1	0
Medical doctor and clinical officer	3	1
Nurse and midwife	25	13
Nurse aide and health assistant	7	2
Others (e.g. lab tech, drug dispenser)	4	1
*Type of health facility*		
Health centre I/II	13	4
Health centre III	20	13
Health centre IV	5	0
Hospital	2	0

At the time of the study, the average time a health worker had served at their current post was 4.5 years (range 1 month to 36 years). Over 90% of the health workers reported that prior to engaging in the microplan development and utilization process, they worked at a different health facility, were at school or were volunteers at the facility. Thus, the microplanning process in specific catchment areas was being implemented by relatively “new” health workers to that area. One third (36%) of health workers at the district-level facilities had not been involved in the microplanning process prior to their current job. Most facilities (95%) reported having a health information system through which they monitored their immunization activities. Slightly more than half (57%) of the facilities had an updated microplan in place. Notably, very few (27%) privately owned health facilities that participate in routine immunization activities had an updated microplan in place, compared to public (71%) and NGO/faith-based organization-owned (60%) facilities. According to National Immunization Programme records reviewed, in Luwero district, the proportion of facilities with updated microplans was reported as 65%, compared to 57% in Kapchorwa district.

### Health workers’ understanding about microplans and the microplanning process

The majority of health workers (*N* = 40) were generally aware of the presence and importance of microplans and the microplanning process in their health facilities. They further acknowledged the significant roles of microplans in identifying and solving several health challenges within communities at the health facility, district and national levels. Overall, while most health workers were aware of the presence and intended benefits of microplans as a means to improve health service delivery, many of them in HC II and III facilities (*n* = 15) had never participated in the microplannning process and did not understand how to implement the microplans.

*Microplanning? I don’t know its meaning. First tell me the meaning of microplanning, then I will answer*. (Nurse at Luwero, Kasozi HC III)

*I have not much information but what I understand deals with taking drugs which are adequate to the health facilities*. (Chemosong Health Centre II, Kapchorwa)

### Catchment area

Notably, some health workers (*N* = 12) at several health facilities admitted that they did not comprehend some of the issues concerning their catchment area. For instance, being unsure of their catchment population emerged as a major hindrance in the development of the microplan.

### Training of health workers

While most health workers mentioned that they were not trained and did not have specific roles in the microplanning process, other health workers within these health facilities had received training on microplanning. Those who received training mostly included facility managers, EPI focal persons, adolescent health focal persons and midwives. As a result, these health workers were often the ones considered to have specific roles and responsibilities in the microplanning process. Participants also added that microplanning training was carried out at different phases, and they were not certain of the training content. Notably, data revealed that some of the training and capacity-building activities were not well coordinated. For example, 23 participants indicated that the process was characterized by various formats including workshops, one-on-one sessions or briefs provided by colleagues who had returned from a training session. Some participants perceived the time allocated to learn about the microplanning process to be insufficient, as stated by this participant:

*I went to a HC (health centre), joined people being oriented and picked a few things on MP (microplan). Most of us were mentored on MP for 2 hours only*. (Nurse at Kapchorwa, Kaserem HC III)

### Information system for construction of microplans

A majority of health workers (*N* = 42) identified various sources of information for the microplanning process including outreach activities, family planning activities, youth-friendly services, population and demographic data for villages, parishes and sub-counties, and records of different health activities. Health workers further cited specific information necessary to construct microplans, such as identifying areas to be prioritized and planned for, ascertaining favourable days for outreaches, classifying facilities to be covered and following the work plan.

*We had first the general map of the service area. We got the target population per month. We also used the distance of the outreach post to the facility. We also allocated the number of staff to go for the outreach, and facilitation for each staff. We also noted the specific mobilizers per post*. (Kapchorwa, Kaserem HC III)

*Every month we look at it to know how many have completed the dose and have defaulted. We refer to it every month to know why some mothers default, why some mothers have not brought their babies*. (Senior Medical Officer, Luwero HC IV)

Despite knowledge about the importance of having information for constructing microplans, health workers revealed that accessing relevant information remains challenging, as most health records are not integrated into the health information management system of health facilities, and sometimes records are misplaced before they can be archived. Additionally, lack of updated information necessary to construct microplans was cited as a major hindrance to the MP process. As a result, there is limited knowledge about communities and programme catchment areas, which greatly affects the planning process. For example, health facilities’ ability to identify hard-to-reach areas and to prioritize areas according to need is limited. The microplanning tool was also perceived to be bulky (15 to 17 pages), complex and difficult to complete. This is in the context of an already overtasked health workforce, especially at the lower-level health facilities.

### Importance of the microplan

All health workers mentioned that microplans are essential for effective service delivery, although some recognized that microplans are more easily planned and implemented at higher-level health facilities like HC IIIs and IVs, because of the presence of more qualified staff, better health equipment and generally a more organized health system.

Some participants perceived that microplans had a significant impact on delivery of health care services, particularly child immunization. Most participants (*N* = 30) mentioned that microplans were helpful for vaccine requisition, obtaining information about new vaccines, providing health education to communities about the importance of immunizing children, and estimating vaccine stock-outs.

*Microplan gave us the number of villages to be covered at the outpost*. (Laboratory Technician at Kapchorwa, Cheptuya HC III)

*From overall populations, we decided, come up with target population per post, per month as well. It helps in ordering the quantity of vaccine according to the number of children*. (Nurse at Kapchorwa, Kaserem HC III)

Some participants also mentioned that the microplan guides health workers on what has to be accomplished and timelines for planned activities, thus enabling them to identify barriers to immunization activities and identify solutions. Such data were reportedly used to avoid vaccine stock-outs and to plan for subsequent health programmes. Additionally, some respondents noted that microplans ease health workers’ duties since they will know when a particular activity is to be implemented, who will implement it, who the target population is and the potential impact of the activity.

…*how you can plan to carry out the activities of immunization so that it can run smoothly to reach the community and to see whether the immunization exercise can reach up to the grassroots*. (Partner NGO Administrator and Member of Health Ministry Technical Working Group on Immunization)

## Discussion

Our findings show that only 57% of the health facilities had updated microplans, and further describe health workers’ perceptions, knowledge and challenges about the microplanning process. Factors that contribute to lack of updated microplans in some facilities include health workers’ limited knowledge about the MP process, especially at the peripheral health facilities, the complex, bulky nature of the MP tool, and inadequate information on the catchment area and catchment population.

Despite having introduced the microplanning tool over 10 years ago, knowledge gaps were identified among health workers who were supposed to be using the tool on a day-to-day basis. The major reasons for the low knowledge include staff attrition, recruitment of new staff and targeting of only specific health workers for training. To bridge the knowledge gap, we propose that future training should include all health workers at the facility and should provide a standard “how-to” guide. The how-to guide should be designed to target the health facility workers at the lower levels, who appeared to face more challenges in adapting their practice and knowledge to optimally utilize the microplan process. In the long term, microplanning should be integrated in the pre-service curriculum so that all newly qualified health workers acquire the knowledge.

Currently, there are efforts to integrate other programmes like maternal health, nutrition and child health into the microplanning tool. In Mongolia, the microplanning tool was shown to have wider health system applications, especially in responding to maternal and child health service needs [11]. Integration of these programs into the immunization microplanning tool would require the involvement of other health workers within the facility, hence reducing the burden of developing the tool by immunization staff. However, we note the potential risk of overburdening the very health workers struggling to adapt the microplan, as a lack of careful integration may make the process more cumbersome. The already overburdened health workers at the lower-level health facilities would need to carefully consider how best to integrate planning processes for other services with the microplan process.

Knowledge of the health facility catchment area is a critical starting point of a good microplan [1, 11, 12]. In this study, health workers considered inadequate information about catchment area and catchment population as a major hindrance to the development and utilization of the microplans. The process of ascertaining the catchment area for each health facility should be initiated at the district level, with involvement of the planning department and local authorities to ensure that all areas are adequately served. The use of geographic information system (GIS)-based maps to clearly demarcate catchment areas can improve the quality of the microplan. Notably, improved vaccination coverage was demonstrated in Nigeria through the use of GIS [13, 14]. Our data further support the need to tailor the microplanning process to the local context to ensure optimal use of microplans [15–17]. In some health facilities where the catchment area was known, clients still preferred to obtain services from other facilities outside the designated catchment area for reasons of convenience. In China, an integrated catchment area approach that takes into account people’s actual behaviours was found to improve accessibility to healthcare facilities when compared to the traditional catchment area methods [18]. The bulkiness and complexity of the microplanning tool was cited as another hindrance in its development and utilization. The tool can be reviewed and reduced from the current 17 pages, while ensuring that content is not lost. The Ministry of Health’s immunization programme staff should devise simpler forms to capture information that is easy to tally at the lower-level health facilities for further compilation at the district and national levels. The tallying process should be intertwined with more frequent support supervision visits by the higher-level health workers.

We should note that although challenges in utilization of the microplan were reported in both districts, Luwero’s immunization coverage was higher than that of Kapchorwa. This may point to the influence of other factors, beyond implementation of the microplan, on routine immunization. We caution that any conclusion about the impact that inability to optimally utilize microplanning may have on low coverage should take into account the potential effect of social and economic differences between the districts. Further research is needed to examine plausible causes of differences in the coverage between the two districts.

## Conclusion

Our findings point to long-standing challenges to the microplanning process that hinder its development and utilization, particularly in rural districts in Uganda. To ensure sustainable training of health workers on the microplanning tool, there is a need for more than one-off sessions, and rather continuous education and support supervision. There is also a need to review the microplan tool to make it easier to utilize, particularly for lower-level, rural-based health workers. For instance, in addition to a less bulky and complex tool, the Ministry of Health should introduce how-to training and guides to standardize the process of microplan development and use. Once properly developed, the microplanning tool can be used to reach every child and effectively improve vaccination coverage.

## Data Availability

The datasets used and/or analysed during the current study are available from the corresponding author on reasonable request.

## Abbreviations

DHT: District health team
DPT3: Diphtheria, pertussis, tetanus, hepatitis B and *Haemophilus* influenza vaccine
EPI: Expanded Programme on Immunization
FGD: Focus group discussion
GIS: Geographic information system
HC I: Health centre level I
HC II: Health centre level II
HC III: Health centre level III
HC IV: Health centre level IV
MP: Microplan
NGO: Nongovernmental organization
PI: Principal investigator
RAs: Research assistants
REC: Reaching Every Community/Child
RED: Reaching Every District
UNCST: Uganda National Council for Science and Technology
UNICEF: United Nations Children’s Fund
WHO ERC: WHO Research Ethics Review Committee
